# Effect of *Carum carvi* essential oil on *ERG6* gene expression and virulence factors in *Candida albicans*

**DOI:** 10.18502/CMM.6.2.3628

**Published:** 2020-06

**Authors:** Samira Nasiri, Masoomeh Shams Ghahfarokhi, Mehdi Razzaghi Abyaneh

**Affiliations:** 1 Department of Mycology, Faculty of Medical Sciences, Tarbiat Modares University, Tehran, Iran; 2 Department of Mycology, Pasteur Institute of Iran, Tehran, Iran

**Keywords:** Antifungal activity, *Candida albicans*, *Carum carvi*, *ERG6*, Virulence factors

## Abstract

**Background and Purpose:**

The present study was conducted to investigate the inhibitory effects of *Carum carvi* essential oil (EO) against *ERG6* gene expression in relation to fungal growth and some important virulence factors in *Candida albicans*.

**Materials and Methods:**

The minimum inhibitory concentration (MIC) of *C. carvi* EO against *C. albicans* was determined by the Clinical and Laboratory Standards Institute M27-A4 method at a concentration range of 20-1280 μg/ml. Furthermore, the expression of *ERG6* gene was studied at the 0.5× MIC concentration of *C. carvi* EO using real-time polymerase chain reaction. The proteinase and phospholipase activities, cell surface hydrophobicity (CSH), and cell membrane ergosterol (CME) content of *C. albicans* were also assessed at the 0.5× MIC concentration of the plant EO using the approved methods. In addition, fluconazole (FLC) was used as a control antifungal drug.

**Results:**

The results indicated that the MIC and minimum fungicidal concentration of *C. carvi* EO for *C. albicans* growth were 320 and 640 μg/ml, respectively. The expression of fungal *ERG6* at an mRNA level and ergosterol content of yeast cells were significantly decreased by both *C. carvi* EO (640 μg/ml) and FLC (2 μg/ml). The proteinase and phospholipase activities were also reduced in *C. carvi* EO by 49.82% and 53.26%, respectively, while they were inhibited in FLC-treated cultures by 27.72% and 34.67%, respectively. Furthermore, the CSH was inhibited in EO- and FLC-treated cultures by 12.75% and 20.80%, respectively.

**Conclusion:**

Our findings revealed that *C. carvi* EO can be considered a potential natural compound in the development of an efficient antifungal agent against *C. albicans*.

## Introduction

The prevalence of infections related to* Candida* species has increased over the past three decades, especially in patients with different conditions, such as those with an immunocompromised state and AIDS, tissue transplantation, antibiotic therapy, and malignancy diseases [ 1 ]. *Candida albicans* is a major pathogenic agent that profoundly invades various parts of the human body by promoting hyphal switching, surface recognition molecules, phenotypic switching, and extracellular hydrolytic enzyme production attributed as virulence factors [ 2 , 3 ]. Extracellular hydrolytic enzymes, such as phospholipases and secreted proteinases, facilitate adherence, tissue penetration, and host invasion [ 2 - 4 ]. 

The adherence of *C. albicans* to host cells is an essential early step in fungal infection related to cell surface hydrophobicity (CSH) [ 2 , 5 ]. *Candida albicans* can also adhere to the surfaces of medical devices and form biofilms. It is well documented that there is a positive association between the degree of virulence and the ability to form infection [ 6 ]. The *ERG6* has been reported to be a putative structural gene for ergosterol biosynthesis and essential for the normal functioning of the fungal cell membrane [ 7 ]. 

Several studies have addressed the pathogenic factors in* Candida* species to facilitate the diagnosis, treatment, and prevention of candidiasis [ 8 - 11 ]. Herbal agents with antifungal activity represent an ideal alternative for chemical medicines regarding the limitations of synthetic antifungal drugs with a high production cost and numerous side effects [ 8 ]. Accordingly, the antibacterial and antifungal effects of many plant species have been widely investigated during the recent two decades [ 12 - 17 ].

*Carum carvi* L. (caraway) is an annual herbaceous plant that belongs to the Umbelliferae family. The plant is native to some certain regions of Iran and is used in foods and herbal medicine [ 18 ]. *Carum carvi* have been well documented as a powerful antifungal and antimicrobial compound in both traditional and modern medicines [ 19 - 21 ]. The present study was conducted to investigate the potential effect of *C. carvi* essential oil (EO) on the growth, proteinase and phospholipase activities, CSH, and cell membrane ergosterol (CME) content of *C. albicans*. This study also involved the evaluation of the effect of *C. carvi* EO on *ERG6* gene expression using real-time polymerase chain reaction (PCR) assay.

## Materials and Methods

***Fungal strain and *Carum carvi* essential oil***

For the purpose of the study, *C. albicans* standard strain ATCC10231 was obtained from the Pathogenic Fungi Culture Collection of the Pasteur Institute of Iran (http://fa.pasteur.ac.ir/VisitDetails.aspx?Id=1311). The strains were cultured on Sabouraud dextrose agar (Merck, Germany) for 48 h at 37°C. In addition, *C. carvi* seeds were purchased from the market. Plant materials were steam distilled for 90 min in a full glass apparatus. The EO was also prepared by the hydrodistillation of sterilized seeds using a Clevenger-type apparatus over a period of 4 h. The yield of EO was about 0.25% and was kept at 4°C [ 21 ].

**Antifungal susceptibility assay**

Antifungal susceptibility assay was conducted according to the guidelines of the National Committee for Clinical Laboratory Standards CLSI M27-A4 method [ 22 ]. Briefly, *C. albicans* was adjusted to a final concentration of 0.5-2.5×10^3^ CFU/mL in RPMI-1640 (Sigma-Aldrich, USA), using 3-(N-morpholino) propanesulfonic acid medium and then added to a 96-well plate. To prepare the stock solution, *C. carvi* EO was dissolved in dimethyl sulfoxide (Sigma-Aldrich, USA) to obtain a final concentration of 100 mg/ml. Subsequently, two-fold serial dilutions of the EO were prepared in RPMI to obtain the final concentrations of 20-1280 μg/ml. 

For fluconazole, the serial two-fold concentrations of 0.5-256 µg/ml were prepared from a stock solution of fluconazole (Sigma-Aldrich, USA) in methanol (Merck, Germany). The plates were incubated at 35°C for 24-48 h. The minimum inhibitory concentration (MIC) of *C. carvi* EO and fluconazole was defined as 100% inhibition of fungal growth in 96-well microplates by visual assay. For determining minimum fungicidal concentration (MFC), 50 µl of the contents of each well over MIC with no visual fungal growth was transferred to Sabouraud dextrose agar (SDA) plates and incubated for 72 h at 35°C. The MFC was defined as the minimum concentration of EO and fluconazole which caused no growth on SDA plates. All tests were conducted in triplicate in three separate experiments.

**Proteinase activity assay**

The determination of proteinase activity was assessed according to Dabiri et al. [ 11 ]. Briefly, 10 µl of a 18-hour yeast suspension (106 cells/ml) was inoculated on bovine serum albumin (1%) medium which contained dextrose (2%), MgSO4 (0.05%), KH2PO4 (0.1%), and agar (2%) with 0.5× MIC concentration of *C. carvi* EO (160 μg/ml) and fluconazole (2 μg/ml). Petri dishes were incubated for 5 days at 35oC and fixed with trichloroacetic acid (TCA, 20%). The zone of the proteolysis around the yeast colonies was measured according to Price et al. [ 23 ]. 

**Phospholipase activity assay**

The phospholipase production was determined by the zone of precipitation around the fungal colony according to Samaranayake et al. using a slightly modified method [ 24 ]. The egg-yolk agar medium consisted of SDA (13 g; Merck, Germany), NaCl (11.7 g), CaCl2 (0.111 g), and 10% sterile egg yolk. Therefore, 10 µl of the yeast suspension (106 cells/ml) was inoculated on the egg-yolk medium with *C. carvi* EO (160 μg/ml) and fluconazole (2 μg/ml). The precipitation zone around the fungal colony was measured after incubation at 35ºC for 72 h.

**Determination of cell surface hydrophobicity**

The effect of *C. carvi* EO on *C. albicans* CSH was measured by the biphasic hydrocarbon-aqueous phase method [ 11 ]. Briefly, 10 µl of yeast suspension (106 cells/ml) was inoculated onto Sabouraud dextrose broth medium with *C. carvi* EO (160 μg/ml) and fluconazole (2 μg/ml) and incubated at 35°C for 48 h. Subsequently, 5 ml of cell suspension was added to sterile glass test tubes. In addition, a test and an untreated control were prepared from the suspending medium and used as blank. In the next stage, 1 ml of xylene was added to each test suspension. 

The test and controls were placed in a water bath at 35°C for 10 min to equilibrate and then vortexed for 30 sec and returned to the water bath for a further 30 min. The lower aqueous phase was carefully transferred to a clean test tube. The absorbance at 520 nm was measured after mixing for 5 sec to suspend any unwanted aggregates. The suspension without xylene was used as a negative control. The hydrophobicity was described as the percentage reduction in the optical density of the test suspension, compared to that of the control. The percentage of hydrophobicity was calculated as follows:

Hydrophobicity (%)=[1−(A_1_/A_0_)] × 100 

**Assay of cell membrane ergosterol content **

The CME content was measured according to Jahanshiri et al. [ 7 ]. *Candida albicans* (10^6^ cells/ml) was inoculated onto Sabouraud dextrose broth medium with *C. carvi* EO (160 μg/ml) and fluconazole (2 μg/ml) and then incubated at 35ºC for 72 h. The fungal cells were harvested by centrifugation at 5,000 rpm for 5 min, washed with distilled water, dried at room temperature, and weighed. In the next stage, 3 mL of 25% alcoholic potassium hydroxide was added to each pellet and vortexed for 1 min.

The suspension was transferred to glass tubes and incubated at 90°C for 1 h in a water bath and cooled at room temperature. Afterward, 1 mL distilled water and 3 mL n-heptane were added and vortexed vigorously for 3 min. After 30 min, the heptane layer was transferred to clear glass tubes and stored at -20°C for 24 h. Then, 1 mL of sterol aliquot was diluted 5 folds in 100% ethanol and scanned at 230-300 nm using an ultraviolet-visible spectrophotometer (Shimadzu, Japan). The presence of ergosterol and late sterol intermediate 24(2*) DHE in the extracted sample resulted in a characteristic four-peak curve. The ergosterol content was calculated as the percentage of the wet weight of the cell using the following calculation: 

% ergosterol + % 24(28) DHE=[(A281.5/290) × F] /fungal pellet weight

%24(28) DHE=(A230/518) × F / fungal pellet weight

% ergosterol=% ergosterol + % 24 (28) DHE − % 24 (28) DHE

where F is the factor for dilution in ethanol and 290 and 518 are the E values determined for crystalline ergosterol and 24(28) DHE, respectively.

**Determination of *ERG6* gene expression**

In order to evaluate the effect of *C. carvi* EO on the expression of *ERG6* in *C. albicans*, total RNA was extracted from the treated fungal at the 0.5× MIC concentration of *C. carvi* EO (160 μg/ml) and fluconazole (2 μg/ml) by the RNX-Plus kit (Sinacolon, Iran). In addition, RNA concentrations and purity were determined, and complementary DNA (cDNA) synthesis was carried out using a kit (Fermentas, USA) following a study performed by Jahanshiri et al. [ 25 ]. The primers used in this study included F: 5'GTGGTGTAGGTGGTCCTGGT 3' and R: 5' CAATGGCATAAACAGCATCG 3' (ERG), as well as F: 5' CCA GCT TTC TAC GTT TCC 3' and reverse: 5' CTG TAA CCA CGT TCA GAC 3' (ACT1) [ 26 ]. To calibrate the real-time quantitative PCR system, the standard curve of the serial dilutions (10−1 to 10−4) of *C. albicans* was drawn with cDNA as a template. Real-time PCR was carried out using the SYBR green master mix (Applied Biosystems) in a final volume of 20 µl reaction, including 10 µl master mix (2X), 1 µl primers (10 mM), 1 µl of total cDNA sample, and distilled water for each reaction. Each sample was normalized for the amount of the template to the level of *ẞ-actin* as a reference gene. The experiments were repeated in triplicate for each sample. The PCR conditions consisted of an initial incubation at 95°C for 10 min, as well as 40 cycles of 15 sec at 95°C, 1 min at 60°C, and 15 sec at 72°C by the ABI PRISM 7500 thermal cycler (Applied Biosystems). To determine the level of *ERG6* expression, the differences between the threshold cycle (CT) of the samples and calibrator was calculated using the following formula: 

(2^−ΔΔCT^)

**Statistical analysis**

All data were analyzed and compared in GraphPad Prism software, version 6.0 (Sandiego, CA) using the ANOVA test. A p-value less than 0.05 was considered statistically significant.

## Results

**Antifungal susceptibility testing**

The MIC and MFC values of *C. carvi* EO were determined and then compared with those of fluconazole against the standard clinical isolate of *C. albicans* (Table 1). Based on the results, the MIC and MFC values of plant EO against *C. albicans* were 320 and 640 μg/ml, respectively. For fluconazole, these values were reported as 4 and 8 μg/ml, respectively.

**Table 1:** Minimum inhibitory concentration and minimum fungicidal concentration values of *Carum carvi* essential oil and fluconazole (μg/ml) for *Candida albicans*

Antifungal compound	Range	MIC	MFC
Essential oil	20-1280	320	640
Fluconazole	0.5-256	4	8

MIC: minimum inhibitory concentration, MFC: minimum fungicidal concentration

**Determination of proteinase and phospholipase activities **

**Figure 1:**
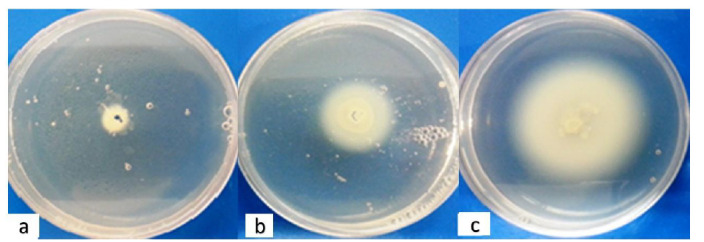
Proteinase activity in *Candida albicans* treated with *Carum carvi* essential oil (a) and fluconazole (b) in comparison to that in the control (c)

**Figure 2:**
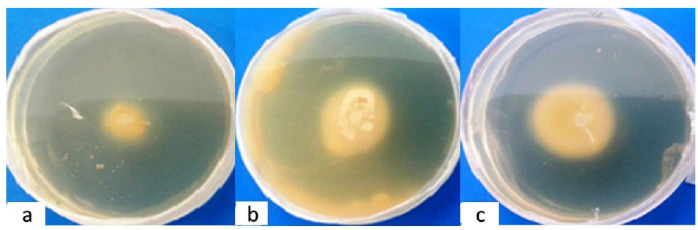
Phospholipase activity in *Candida albicans* treated with *Carum carvi* essential oil (a) and fluconazole (b) in comparison to that in the control (c)

The production of proteinase and phospholipase was reduced in *C. carvi* EO and fluconazole-treated culture (figures 1 and 2). The proteinase and phospholipase activities were reduced in *C. carvi* EO by 49.82% and 53.26%, respectively, while they were inhibited in fluconazole-treated cultures by 27.72% and 34.67%, respectively (Table 2). A significant difference was shown between the treated and control groups in terms of this variable (*P*<0.05). The results revealed that *C. carvi* EO was as effective as fluconazole in the reduction of both proteinase and phospholipase production.

**Table 2:** Determination of proteinase and phospholipase production, cell surface hydrophobicity, and cell membrane ergosterol content in *Candida albicans* treated with *Carum carvi* essential oil and fluconazole

Groups	Proteinase production (mm)	Proteinase inhibition (%)	Phospholipase production (mm)	Phospholipase inhibition (%)	CSH (%)	CME (µg/g)	CME Inhibition (%)
*C. carvi* EO -treated	1.43±0.07	49.8	0.93±0.02	53.26	12.75	0.084±0.01	25.74
Fluconazole -treated	2.06±0.05	27.72	1.30±0.08	34.67	20.80	0.064±0.01	43.36
Control (Non-treated)	2.85± 0.15	00.00	1.99±0.10	00.00	38.98	0.113±0.01	00.00

Results of the mean±SD of two experiments, each in triplicate.

CSH: cell surface hydrophobicity, CME: cell membrane ergosterol

**Determination of cell surface hydrophobicity**

The CSH of *C. albicans* treated with *C. carvi* EO and fluconazole is shown in Table 2. According to the results, the CSH of *C. albicans* treated with *C. carvi* EO and fluconazole respectively reduced by 12.75% and 20.80% in comparison to that of the non-treated control. A significant difference was shown between the treated and control groups in this regard (*P*<0.05). 

**Determination of cell membrane ergosterol content **

The CME contents of *C. albicans* in *C. carvi* EO- and fluconazole-treated cultures were measured at 0.084 and 0.064 µg/g, respectively (Table 2), indicating a significant difference between the treated and control groups (*P*<0.05). The CME was inhibited at the rates of 25.74% and 43.36% in *C. carvi* EO- and fluconazole-treated cultures, respectively. The results showed that fluconazole was more efficient than *C. carvi* EO in reducing the ergosterol content of *C. albicans*.

**Determination of *ERG6* gene expression **

**Figure 3:**
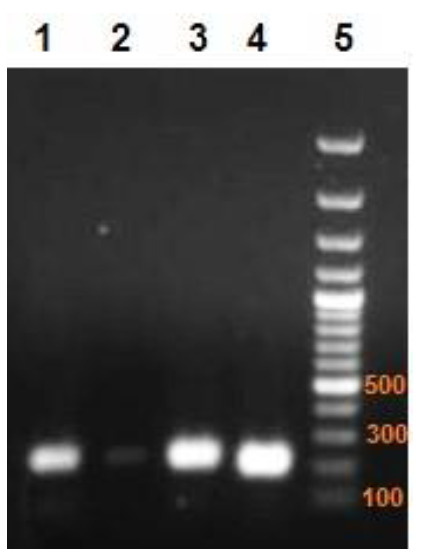
Real-time polymerase chain reaction product electrophoresis showing the presence of complementary DNA bands; 1) *Carum carvi* essential oil-treated sample, 2) negative control (D.W.), 3) fluconazole-treated sample, 4) reference gene ACT1, and 5) DNA ladder (100 bp)

The expression of *ERG6* gene, as a mediator for the biosynthesis of ergosterol, was studied by means of quantitative PCR using specific primers in *C. albicans*-treated with 0.5× MIC of *C. carvi* EO (160 μg/ml) and fluconazole (2 μg/ml). The amplification products were also analyzed by agarose gel electrophoresis (Figure 3).

**Figure 4:**
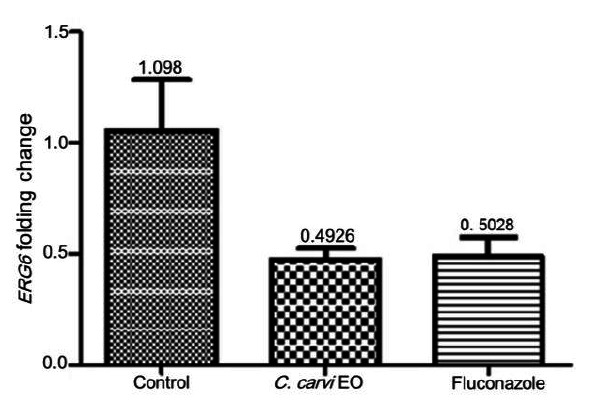
Expression of *ERG6* at the mRNA level in *Candida albicans* treated with *Carum carvi* essential oil (160 μg/ml) and fluconazole (2 μg/mL) (*P*<0.05)

The relative gene expression of *ERG6* was measured according to the [2^−ΔΔCT^] formula. The *ERG6* gene expression in the *C. carvi* EO- and fluconazole-treated samples was reduced by around 2 folds at the mRNA level which was significant in comparison with that in the non-treated control (Figure 4; *P*<0.05). Therefore, both plant EO and fluconazole similarly inhibited *ERG6* expression. Therefore, there was no significant difference between the EO- and fluconazole-treated samples in terms of the inhibition of *ERG6* expression (*P*>0.05).

## Discussion

*Candida albicans* is an opportunistic fungus accounting for a growing number of life-threatening infections in immunocompromised individuals [ 1 , 2 , 27 ]. It has been shown that phospholipase and proteinase production and hydrophobicity may play significant roles in the pathogenesis and virulence of *C. albicans* [ 3 , 7 , 28 ]. Nowadays, many investigations are targeted toward finding new natural products with strong antifungal effects to be used as an alternative for the treatment of fungal diseases. There are several studies addressing the antifungal effects of many organic and natural compounds against *C. albicans* [ 13 - 17 , 20 , 29 ]. *Carum carvi* is known as ‘Black Zira’ in Iran and has been used for many therapeutic purposes in traditional medicine [ 12 , 18 , 19 ]. 

The present study was aimed to investigate the inhibitory effects of *C. carvi* EO against some virulence factors, such as the production of proteinase and phospholipase, CSH, cell membrane ergosterol content, and expression of *ERG6* gene as a mediator for the biosynthesis of ergosterol in *C. albicans*. The EOs are complex natural mixtures composed of numerous constituents at quite different concentrations. Therefore, it is difficult to attribute the antifungal activity to a specific component of this product. 

Carvone (44.5-95.9%) and limonene (1.5-51.3%) have been reported as the main components of *C. carvi* EO. The other constituents include β-myrcene (0-0.4%), trans-dihydrochalcone (0-0.5%), trans-caveolae (0-0.2%), α-pinene, sabinene, n-octanal, trans-β-ocimene, and γ-terpinene. Additionally, cuminaldehyde (22.08%), c-terpinene (17.86%), c-terpinene-7-al (15.41%), p-cymene (7.99%), myristicin, and dillapiol have been identified as the important constituents of *C. carvi* EO [ 21 , 30 , 31 ]. 

The antifungal activity of *C. carvi* EO was studied against a wide variety of fungi [ 20 , 32 - 37 ]. *Carum carvi* EO was found to possess remarkable antifungal activity against* Candida krusei* ATCC 6258,* Candida parapsilosis* ATCC 22019, *Aspergillus niger, Aspergillus flavus,* and *Aspergillus fumigatus*, as well as an effective inhibition against *C. krusei* growth (42-mm inhibition zone) [ 32 ]. *Carum carvi* reportedly acts as an inhibitor of aflatoxin production in A. parasiticus. However, it showed no effect on fungal growth even at the highest concentration (1000 μg/ml) [ 21 ]. 

Our results showed that *C. carvi* EO at a concentration of 320 μg/ml declined the growth rate of *C. albicans* by 50%. However, at the concentration of 640 μg/ml, this product completely inhibited fungal growth. With respect to the MIC values of fluconazole (4 μg/ml), it had a potency that was 80 times more than that of *C. carvi* EO, which is usual due to the fact that we compared the MIC of a pure antifungal agent with the MIC of a mixed unpurified plant EO. This means that a comparison will be more accurate if the MIC of fluconazole is compared with the MIC of the inhibitory bioactive component of *C. carvi* EO. 

*Carum carvi* EO contains rich oxygenated compounds, such as carvone and limonene, affecting the integrity of the fungal cell membrane. It has been shown that free-terpene hydrocarbons containing oxygenated compounds with low molecular weights are able to penetrate through the cell membrane after combining with lipophilic molecules [ 36 ]. Furthermore, phenolic compounds with OH group and aromatic structure have been identified to contribute to the deactivation of fungal enzymes [ 36 ]. Our findings revealed that *C. carvi* EO could successfully inhibit extracellular proteinase and phospholipase activities in *C. albicans*. Moreover, based on the evidence, proteinase and phospholipase activities are also involved in adhesion and tissue invasion by *C. albicans* [ 4 , 10 ]. The reduction of proteinase and phospholipase activities may be regarded as a consequence of targeting the active sites of these enzymes by *C. carvi* EO. 

Patel et al. observed that a crude extract of Dodonaea viscosa was not able to inhibit proteinase and phospholipase activities in *C. albicans*. However, the presence of phytosterol and tannins in the extract may have caused extensive cellular damage, including cell wall damage [ 9 ]. It was revealed that the susceptibility or resistance of a fungus to the antifungal activity of an EO depends on the capacity of their main compounds. In this study, the presence of *C. carvi* EO (0.5×MIC) caused a significant reduction in CSH similar to fluconazole. 

Ergosterol is one of the main parts of the fungal cell membrane and is reported to be the main target for antifungal drugs. Ergosterol biosynthesis is mediated by ERG gene family among which *ERG6* is an important gene. This gene encodes C-24 methyltransferase, which converts zymosterol to fecosterol in the ergosterol biosynthesis pathway. The *ERG6* gene is required for the normal function of the cell membrane in *C. albicans*; therefore, the mutation of this gene can inhibit the antifungal activities of drugs [ 37 ]. In the current study, CME content was reduced to 25.74% at a 0.5× MIC concentration of *C. carvi* EO (160 μg/ml). In addition, the expression of *ERG6* was decreased around 2 folds at the mRNA level at the same concentration. 

Since the absence of a functional sterol methyltransferase could induce cell hypersensitivity to exogenous compounds, the inhibition of *ERG6* gene expression may increase the potency of new or existing antifungals. Therefore, the natural inhibitors of *ERG6* gene product, such as *C. carvi* EO, introduced in the present study allows for the clinical treatment of candidiasis in a safer manner at a lower dosage [ 26 ]. On the other hand, it has been shown that *C. carvi* EO suppresses the expression of cytochrome P450 1A1 (CYP1A1) at the transcription level [ 38 ], which is responsible for the hepatotoxic effects of azole antifungal agents [ 39 ]. 

## Conclusion

As the findings indicated, *C. carvi* EO could suppress *ERG6* gene as a crucial gene in ergosterol biosynthesis in *C. albicans* and efficiently inhibit the major virulence factors of the fungus, including exoenzymes, cell membrane hydrophobicity, and CME content. The main antifungal components of *C. carvi* EO may be useful as natural alternatives for antifungal drug formulations to manage fungal infections caused by *C. albicans*.
